# Cellular Senescence as a Therapeutic Target for Age-Related Diseases: A Review

**DOI:** 10.1007/s12325-020-01287-0

**Published:** 2020-03-17

**Authors:** Mateo Amaya-Montoya, Agustín Pérez-Londoño, Valentina Guatibonza-García, Andrea Vargas-Villanueva, Carlos O. Mendivil

**Affiliations:** 1 School of Medicine, Universidad de Los Andes, Bogotá, Colombia; 2 Endocrinology Section, Department of Internal Medicine, Fundación Santa Fe de Bogotá, Bogotá, Colombia

**Keywords:** Aging, Non-communicable diseases, Senescence, Senolytics, Therapeutics

## Abstract

Life expectancy has increased substantially over the last few decades, leading to a worldwide increase in the prevalence and burden of aging-associated diseases. Recent evidence has proven that cellular senescence contributes substantially to the development of these disorders. Cellular senescence is a state of cell cycle arrest with suppressed apoptosis and concomitant secretion of multiple bioactive factors (the senescence-associated secretory phenotype—SASP) that plays a physiological role in embryonic development and healing processes. However, DNA damage and oxidative stress that occur during aging cause the accumulation of senescent cells, which through their SASP bring about deleterious effects on multiple organ and systemic functions. Ablation of senescent cells through genetic or pharmacological means leads to improved life span and health span in animal models, and preliminary evidence suggests it may also have a positive impact on human health. Thus, strategies to reduce or eliminate the burden of senescent cells or their products have the potential to impact multiple clinical outcomes with a single intervention. In this review, we touch upon the basics of cell senescence and summarize the current state of development of therapies against cell senescence for human use.

## Key Summary Points

**Table:** 

Aging-associated diseases as a group are the main cause of morbidity and mortality worldwide.
Cellular senescence is a state of replicative arrest, suppressed apoptosis, and a typical secretory phenotype that accompanies aging.
Evidence from mouse models suggests that clearance of senescent cells improves health span and life span, limiting age-associated organ dysfunction.
Several small-molecule compounds (senolytics) have proven to lead to senescent cell clearance, and some of them are being tested in human trials.

## Introduction

Life expectancy for most people born at the beginning of the nineteenth century was between 30 and 40 years, but thanks to social and technological advances, reduced fertility rates, and improvements in survival, humans can now expect to live more than twice as long as their ancestors [1, 2]. Over the recent years, global life expectancy at birth for men and women increased from 66.5 years in 2000 to 72.0 years in 2016 [3]. The number of people aged 60 or older more than doubled between 1980 and 2017 (from 382 million to 962 million), and people within this age group are projected to reach 2.1 billion by 2050. The figure for people over 80 is expected to triple by the same year [2].

Except for lower respiratory infections and tuberculosis, the top ten causes of death among people over 60 are all chronic and non-communicable (ischemic heart disease, stroke, chronic obstructive pulmonary disease, respiratory system cancers, diabetes mellitus, kidney diseases, cirrhosis of the liver, and liver cancer) [4]. For people over 70, Alzheimer’s disease and other neurodegenerative conditions reach 4th place, after being in 14th position as recently as in 2000 [4]. During the year 2017, it is estimated that 80% of all disability was caused by non-communicable diseases [5]. A common biological phenomenon underlying the development of all these chronic, degenerative diseases is cellular senescence [6]. In this article we will review the demonstrated and potential relevance of cellular senescence as a target for age-related chronic diseases. This article is based on previously conducted studies and does not contain any studies with human participants or animals performed by any of the authors.

## What is Cellular Senescence?

Cellular senescence has been known for more than 60 years, but more recently has been classified as developmentally programmed senescence, stress-induced premature senescence, or replicative senescence. In early studies of primary culture, explanted human cells where thought to be immortal and their lack of continuous replication was considered a methodological error [7]. In this context, Hayflick and Moorhead described in 1961 three different growth phases of cultured fibroblasts derived from human fetal tissue [8]. Phase I represented the early growth phase, in which the primary culture was formed. Phase II was characterized by a rapid and sustained cell division which lasted around 10 months until phase III started. In this last phase, bizarre nuclear forms were seen, and mitotic activity progressively decreased until it eventually ended. This last unexpected finding was called the “phase III phenomenon” which was the first description of cellular senescence [8]. Since then, cell senescence has been defined as an induced, irreversible state of cell cycle arrest associated with morphologic changes, apoptosis resistance, and a wide secretory profile known as senescence-associated secretory phenotype (SASP) [6].

As a result of its inducible nature, cell senescence has been conceived as a stress response mechanism against potentially noxious stimuli such as DNA damage, oxidative stress, and oncogene expression, among others [9, 10]. These signals activate several cell cycle regulating processes that prevent proliferation of damaged and potentially malignant cells [11]. Broadly speaking, the most important effectors of cell senescence are the p53/p21 and p16/Rb pathways [12, 13], mostly through their regulation of the G1 phase of the cell cycle [14, 15]. Cellular senescence and quiescence are both forms of growth arrest, yet they differ in several important aspects: Quiescence usually originates from a lack of nutrition or mitogenic signals and is invariably reversible once these nutrients or signals are available again [16]. In contrast, cellular senescence in general persists even after removal of the inducing stimulus, albeit senescent cancer cells may be the exception to this rule [17, 18].

Apoptosis, on the other hand, is a form of programmed cell death in response to severe genomic or metabolic disruption. It is important to realize that apoptosis and cell senescence are entirely different processes; in fact, resistance to apoptosis is a hallmark of cell senescence [19]. Multiple factors such as the extent of DNA damage or oxidative stress, as well as cell type, determine whether cell senescence or apoptosis will occur [20]. Effectors of the combined senescence/apoptosis resistance response include members of the B cell lymphoma 2 (Bcl-2) family, ephrins, phosphoinositide 3-kinases (PI3Ks), forkhead box protein O4 (FOXO4) transcription factors, and the chaperone heat shock protein 90 (HSP90) [21].

The SASP consists of a wide range of proteins encompassing pro-inflammatory cytokines, chemokines, growth factors, and proteases [22, 23]. The main effect of these molecules is the maintenance and propagation of the senescent phenotype through paracrine and autocrine mechanisms [24, 25]. Cell senescence and the SASP play a fundamental role in physiological wound healing [26], tissue repair [27], and embryonic development [28]. However, a persistent, unregulated SASP is involved in the development of age-related diseases. This effect is thought to occur through the induction of a chronic, low grade, sterile inflammatory state that has been dubbed inflammaging [29]. Furthermore, the progressive accumulation of senescent cells is considered a hallmark of aging [30]. Additionally, the SASP has been associated with tumorigenesis [31], epithelial-to-mesenchymal transition [32], and metastasis [33].

Despite these general features, cell senescence is a complex, dynamic, multistep phenomenon [34]. First, cell senescence can be classified as acute or chronic in vivo [35]. Acute cellular senescence refers to the transient effect observed during wound healing and fetal development, in which SASP factors are beneficial. On the other hand, chronic cellular senescence is caused by prolonged exposition to senescence inductors, and in this context SASP factors produce the known detrimental effects. Second, as it has been recently reviewed by Faget et al. [36], the exact composition of the SASP is dependent on both the stimulus and cell type. This may partially explain the different and in some cases even opposite effects of cell senescence and should be acknowledged when approaching senolytic interventions.

## What Causes Cellular Senescence?

There are multiple defined causes of cellular senescence. There are multiple defined causes of cellular senescence. Telomere shortening is a kind of DNA damage that triggers a DNA damage response (DDR), leading to arrested cell cycle in an attempt to repair such damage [37]. The DDR is a group of signaling pathways that coordinate repair activities after DNA damage is detected. Some lesions are subject to direct reversal, but most of them are fixed through catalytic events mediated by multiple enzymes. It has been shown that human cells do not express sufficient telomerase to fully counteract telomere shortening. Eventually, this damage can be recognized as double-strand breaks which leads to chronic DDR activation, leading cells to enter apoptosis or cell senescence [38]. The presence of DNA double-strand breaks is revealed by the occurrence of phosphorylated histone γ-H2AX foci (γ-foci) in their vicinity [39]. These γ-foci are characteristic of cells from aged animals, and of senescing human cells in vitro [40].

**Fig. 1:**
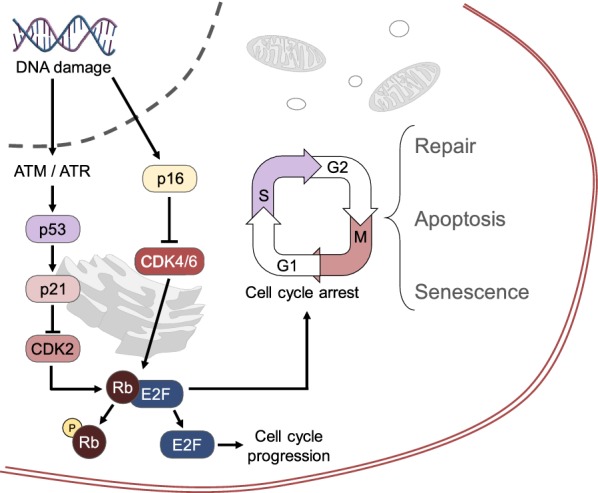
Pathways leading from the DNA damage response (DDR) to cell senescence. E2F is a transcription factor that stimulates the transition from G1 to S phase of the cell cycle. The retinoblastoma protein (Rb) is normally complexed with E2F, inhibiting it and preventing mitosis. When Rb is phosphorylated by a cyclin-dependent kinase (CDK), it releases E2F and cell division ensues. DNA damage activates the ATM or ATR kinases, stabilizing p53 and subsequently stimulating the expression of the protein p21, which is an inhibitor of the cyclin-dependent kinase CDK2. When CDK2 is inhibited by p21 as a consequence of the DDR, Rb remains complexed with E2F, and the cell enters a state of apoptosis or senescence. The DDR also leads to enhanced expression of p16, which inactivates CDK4 and CDK6, also preventing Rb phosphorylation and cell cycle progression

As previously mentioned, a common characteristic of senescent cells is their state of growth arrest, controlled by the p53 and p16/Rb tumor suppressor pathways and intended as an anticancer mechanism. These gatekeeper tumor-suppressing mechanisms prevent both cancer-induced apoptosis and tumor proliferation by inducing permanent cell cycle arrest [41]. The DDR initiates cell senescence through activation of the ATM or ATR kinases, which block cell cycle progression by stabilizing p53 and activating the cyclin-dependent kinase inhibitor p21 [35] (Fig. 1).

Simultaneous proliferative and antiproliferative signals are also associated with cell senescence [42]. When cells arrested by DNA damage and p21 expression are exposed to mitogenic stimuli, they lose their proliferative potential, but also fail to apoptose [42]. One example of such opposing stimuli is high-level RAS signaling, which provides a proliferation signal while also activating CDKN2a locus via “replication stress” [43].

Another known cause of cellular senescence is endoplasmic reticulum (ER) stress due to the accumulation of unfolded/misfolded proteins in the ER lumen. Cells have two systems to avoid the accumulation of unfolded proteins: ER-associated protein degradation (ERAD) and the unfolded protein response (UPR) [44]. The UPR restores ER protein homeostasis by attenuating protein synthesis and activating transcription factors that regulate genes encoding for chaperones, components of the autophagy system, and components of the ERAD system. ER overactivity could be an inductor of senescence-associated oxidative stress, but it is still unclear whether ER stress is a cause of cell senescence or a feature of it [44].

**Fig. 2:**
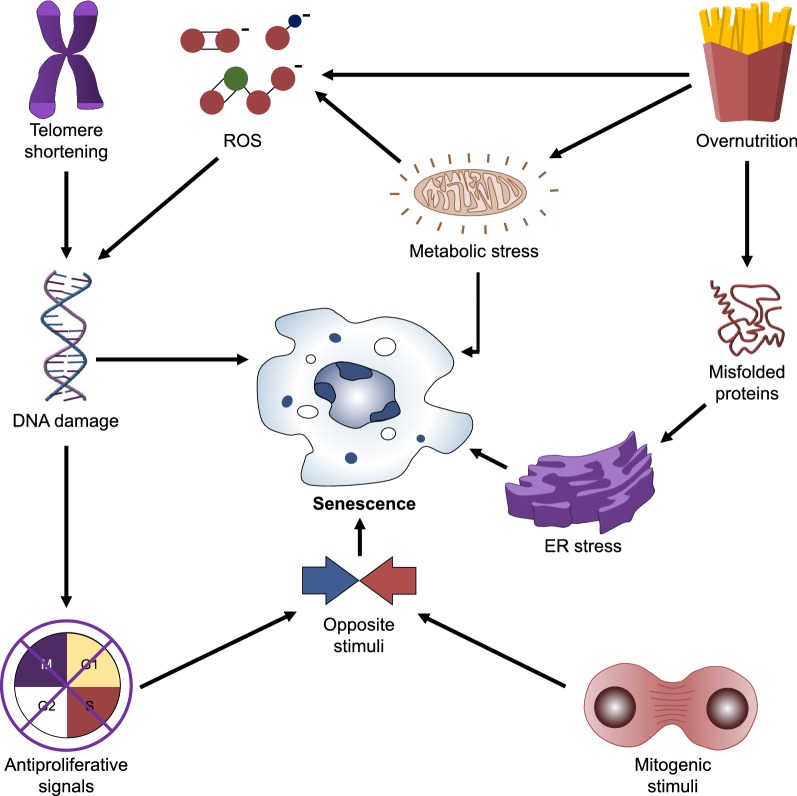
Triggers of cellular senescence

Overnutrition and metabolic stress may also lead to cell senescence because they disrupt the cellular capacity to transport or process macronutrients [45]. Adipose tissue from obese mice shows enhanced activity of senescence-associated β-galactosidase (SA-β-Gal) and higher expression of p53, both associated with the senescent phenotype [46]. Obese individuals display increased production of reactive oxygen species (ROS) at adipose tissue [47]. These increased ROS may in turn induce cell senescence by a process involving the DDR, epigenetic regulation, and protein secretion patterns controlled by p53, p21, and Rb [48]. Figure 2 summarizes the triggers of cellular senescence.

## How are Senescent Cells?

**Fig. 3:**
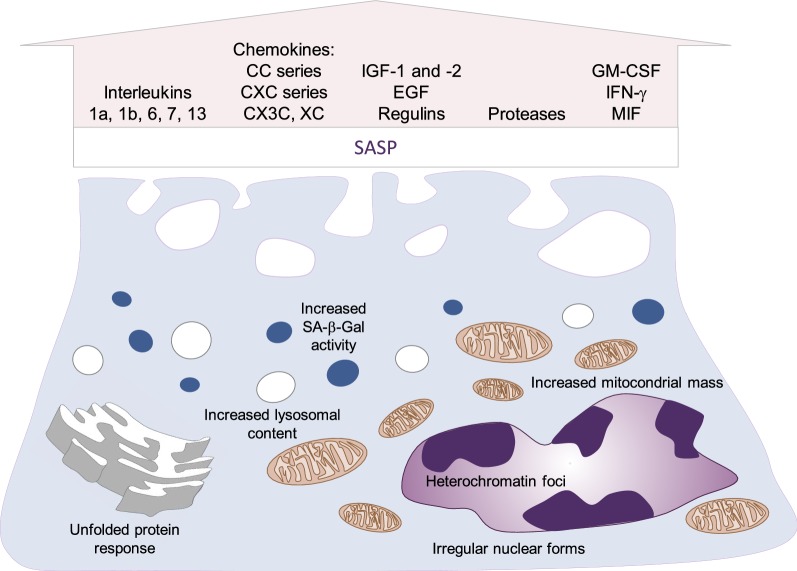
Characteristics of senescent cells and the senescence-associated secretory phenotype (SASP). EGF epidermal growth factor, IGF insulin-like growth factor, GM-CSF granulocyte-macrophage colony-stimulating factor, IFN-γ interferon gamma, MIF macrophage migration inhibitory factor

Senescent cells in vitro are characterized by specific morphological features: They are enlarged, flat, vacuolated, and in some cases multinucleated with irregular nuclear forms [49]. Causes of this altered appearance include cytoskeleton abnormalities [50, 51] and increased organelle number or size. Specifically, the most affected structures are mitochondria, ER, nucleus, and lysosomes [52]. It has been hypothesized that these alterations are attributable to the influence of growth factors (secreted within the SASP), which increase organelle mass as preparation for cell division, but as a result of the cell cycle inhibition result in organelle accumulation. Interestingly, the augmented lysosomal content of senescent cells is the basis for one of the most important assays for their identification: SA-β-Gal activity [34]. β-Galactosidase is found within lysosomes, where an acidic pH (4.0–4.5) is required for maximal enzymatic activity. However, SA-β-Gal activity is tested at pH 6, an unfavorable condition that reduces enzymatic activity by almost 99% [53]. In this scenario, only cells with increased lysosomal content and therefore increased lysosomal β-galactosidase show positive staining, allowing histologic identification of senescent cells (Fig. 3).

Pro-inflammatory cytokines represent the main component of SASP. Among the interleukins associated with cell senescence, the most studied are IL-6 and IL-1. The former is a classical inflammatory interleukin associated with different inflammatory diseases like rheumatoid arthritis and some types of cancer [54]. Once IL-6 binds its receptor, a complex is formed with two GP130 molecules, leading to activation of the JAK-STAT signaling pathway [55]. IL-6 is secreted by senescent cells and plays a fundamental role in the paracrine and autocrine maintenance of the SASP, as a direct inductor of cell cycle arrest and as a pro-mitogenic factor in oncogene-induced cell senescence [56]. On the other hand, both IL-1α and IL-1β are secreted by and expressed at the surface of senescent cells [57]. They further extend the SASP profile by promoting NF-κB and C/EBPβ DNA-binding capacity and promoting IL-6 and IL-8 secretion [58].

Similarly, several chemokines are overexpressed and secreted by senescent cells. These molecules are classified according to the presence of cysteine (C) and other amino acids (X) residues at their N-terminal domain. The main four types are CC, CXC, XC, and CX3C [59]. Additionally, a letter L or R is added to the name depending on whether it is the ligand (L) or the receptor (R) that is been referred to. Several CC and CXC chemokines are secreted by senescent cells: Monocyte chemoattractant proteins (MCP)-1, -2, -3, -4 (corresponding to CCL-2, -7, -8, and -13), macrophage inflammatory protein (MIP) 1α and 3α (CCL-3, -20), monotactin-1 (CCL-16), and IL-8 (CXCL-8), among others [60]. Of clinical interest, lymphotactin (XCL-1) is secreted along with other chemokines by aged CD4^+^ and CD8^+^ T cells and is thought to participate in T cell chemokine-dependent diseases of aging like rheumatoid arthritis and atherosclerosis [61]. The only known CX3C chemokine, fractalkine (CX3CL-1), is overexpressed in senescent biliary epithelial cells and it is thought to exacerbate ductal inflammation in primary biliary cholangitis [62]. In the central nervous system, fractalkine is a fundamental mediator of the communication between glial cells and neurons. Of note, this chemokine is decreased in aged hippocampal cells [63] and has been attributed both beneficial and deleterious roles in the aging brain and neurodegenerative diseases [64].

Growth factors have also been described as components of the SASP. Insulin-like growth factors (IGFs) 1 and 2 (IGF-1, IGF-2), several epidermal growth factor receptor (EGFR) ligands (epiregulin, amphiregulin), and the endothelial growth factor (EGF) are major examples [60]. In particular, IGFs have been extensively studied in this context, as IGF signaling is involved in energy metabolism, embryogenesis, and even neoplasia [65]. Related to cellular senescence, IGF-1 has shown a dynamic function in cultured fibroblasts: Acute exposition promotes cell proliferation, while prolonged exposition promotes senescence by p53 activation via SIRT1 deacetylase inhibition [66].

Alongside cytokines and grow factors, several extracellular enzymes and associated proteins are part of the SASP. The most representative groups involved are matrix metalloproteinases (MMPs), tissue inhibitors of metalloproteinases (TIMPs), and serine proteases [67] (Fig. 3). Physiologically, the integrity of the extracellular matrix (ECM) is maintained by tightly controlled degradative and synthetic processes mediated respectively by MMPs and TIMPS [68]. Senescent cells perturb this balance by secreting a wide range of MMPs [32, 69–72], which may be involved in the development of age-related diseases ranging from dermal collagen damage [73] to enzymatic atheroma plaque destruction [74]. It is known that besides ECM components, MMPs cleave inflammatory and immune mediators like chemokines. For example, MMP-1, -2, -3, -8, and -13 cleave CCL-2, -7, -8, and -13, resulting not only in the degradation of the CC chemokines but also in the formation of several CCR antagonists [75]. Whether this can be regarded as a regulating mechanism of the inflammatory response or as a strategy to reduce immune clearance of senescent cells [67] is still unclear.

As explained above, a vast array of environmental stimuli incite cell cycle arrest and play a role in the induction of cell senescence. Cyclin-dependent kinases (CDKs) phosphorylate key regulators of mitosis, determining cell cycle progression or arrest. The main regulators of the transition from G1 to S phase of cell cycle are encoded by the CDKN2A/p16^INK4A^ locus; hence, expression of this gene is used as a marker of cell senescence [76]. The protein p16 binds the CDK4/6 complex, inhibiting its kinase activity and preventing the phosphorylation of multiple downstream targets, including the retinoblastoma (Rb) protein. Unphosphorylated Rb is associated with the E2F1 transcription factor, blocking its translocation to the nucleus and therefore the transcription of target genes involved in the G1/S phase transition [77] (Fig. 1). Another molecule involved in cell cycle arrest is the p53-induced protein p21, which acts as a potent kinase/cell cycle inhibitor, but also as an anti-apoptotic molecule, having both pro- and antiproliferative features [78].

## What are the Functions of Cellular Senescence?

**Fig. 4:**
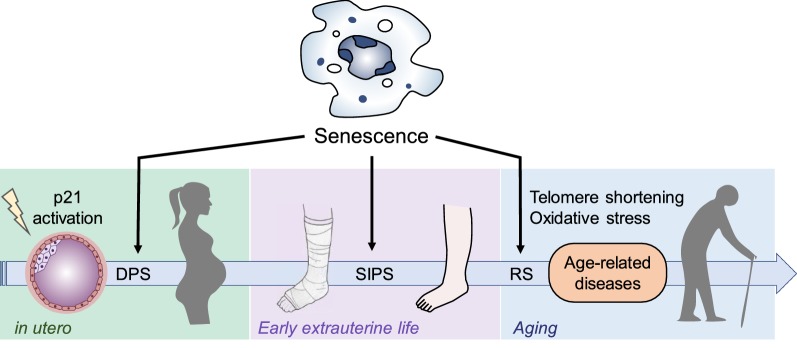
Functions of cellular senescence throughout the life span. DPS developmentally programmed senescence, SIPS stress-induced premature senescence, RS replicative senescence

Cellular senescence has a pleiotropic effect that depends on the trigger, the cell type involved, and the hormonal background [79]. The study of cellular senescence has been addressed in three contexts: developmentally programmed senescence (DPS), stress-induced premature senescence (SIPS), and replicative senescence (RS) [80] (Fig. 4).

### Developmentally Programmed Senescence (DPS)

Over the last 10 years, evidence on the involvement of cell senescence during early development has been mounting. Embryonic developmental structures of mammalians express cell senescence biomarkers, but in contrast to adult cells they mostly employ p21 as cell senescence effector and DNA damage is not required for the response to occur [28, 81]. DPS can be thought of as a primitive form of cell senescence [28]. On the other side, senescent natural killer cells (NK) facilitate embryonic implantation [82]. NK cells are the most abundant immune cells at the maternal/fetal interphase during early pregnancy [83]. The fetal trophoblast activates the CD158d receptor in senescent NK cells, initiating the p21 signaling pathway and inducing a secretory proteome that promotes vascular remodeling and angiogenesis [84].

### Stress-Induced Premature Senescence

After birth, cells are exposed to multiple stressors that may activate a more evolved pathway known as stress-induced premature senescence (SIPS) [80]. This pathway is initiated after an external stimulus triggers cell cycle arrest [85] and plays an important role in physiological processes like wound closure. In response to tissue damage, senescent cells secrete platelet-derived growth factor AA (PDGF-AA) [26] and the extracellular matrix-associated signaling protein CCN1 to accelerate healing [86]. Nevertheless, senescent cells can also produce proteins that promote stemness or reduce reparative capacity [i.e., plasminogen activator inhibitor-1 (PAI-1)] [34].

### Replicative Senescence

This model is best illustrated by the cellular response to a finite replicative capacity [7, 80]. Throughout aging, progressive telomere shortening and decreased senescent cell clearance generate tissue and organ dysfunction [8]. In vivo, the first unequivocal evidence of a link between cell senescence and aging was the resolution of age-associated disorders after selective apoptosis of cells expressing p16 in a progeroid mouse model [87].

The impact of the replicative senescence of cells has been proven in aging-associated disorders. Vascular smooth muscle and endothelial cells from atherosclerotic plaques express CDKN2A/B and induce a SASP in plaque macrophages, contributing to plaque instability, elastic fiber degradation, and fibrous cap thinning [88]. There is conflicting evidence about the effect of senescent cells on glucose metabolism. Humans with hypomorphic alleles of the *CDKN2A* gene have an enhanced capacity for pancreatic insulin secretion [89], but overexpression of INK4a (the mouse ortholog of CDKN2A) increases peripheral insulin sensitivity in mice [90]. Meanwhile, clearance of senescent cells has been associated with less chemotherapy-induced asthenia [91]. There is limited evidence of a causal association between cell senescence and neurodegeneration, but neuroinflammation is a known feature of Alzheimer’s and Parkinson’s diseases [92]. Brain cortex from patients with Alzheimer’s disease and mouse models of the disease display increased expression of cell senescence markers [93, 94]. Finally, oncogenesis activates cell senescence as a tumor-suppressive response to inhibit malignant transformation. An example is the ability of mutations in the oncogenes *KRAS, BRAF, PTEN*, and *NF1* to trigger cell senescence [92, 95]. In advanced stages of cancer though, senescent cells may actually promote tumor progression through the SASP [96].

## What is the Impact of Cellular Senescence on Health?

There is abundant evidence demonstrating the progressive accumulation of senescent cells associated with multiple age-related diseases. For a comprehensive review of this evidence, we suggest references [92, 96]. The profound influence that senescent cells have on the function of complete organisms has been proven using different genetic approaches. The ATTAC (apoptosis through targeted activation of caspase-8) transgenic mouse model is used to study the effects of the removal of a specific cell type in vivo. Initially developed for the ablation of mature adipocytes [97], ATTAC involves the transgenic introduction of a construct containing a caspase-8/FKBP complex, whose transcription is under the control of a promoter that is active only in the targeted cell population (in adipocytes, the *Fabp4* promoter) [97]. A further elaboration of this technique is the FAT-ATTAC model, in which apoptosis is induced through treatment with AP20187, a molecule that induces dimerization of two half-FKBP/caspase-8 chimera fragments [98], with subsequent caspase-8 activation and apoptosis, clearing the target cells.

**Fig. 5:**
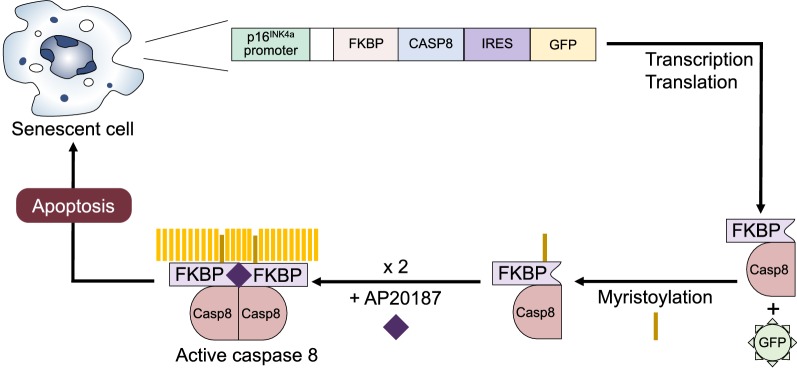
Mechanism for the inducible apoptosis of senescent cells in INK-ATTAC mice. Zygotic cells are transfected with a construct encoding an FKBP/Casp8 complex plus a green fluorescent protein, all under the control of the *Ink4a/Arf* promoter (active only in senescent cells). Administration of the dimerizer AP20187 leads to conformation of an active caspase-8 and selective apoptosis of senescent cells in the whole organism. FKBP FK506 binding protein, IRES internal ribosomal entry site, GFP green fluorescent protein

Baker and colleagues introduced the INK-ATTAC transgenic model, inspired by the FAT-ATTAC but aimed at the elimination of senescent cells. In INK-ATTAC, the FKBP-CASP8 transgene includes also a green fluorescent protein, and the whole construct is under the control of the *Ink4a/Arf* promoter, which is only active in senescent cells [99, 100]. After the administration of AP20187 (AP), these mice ablate naturally occurring p16^Ink4a^ (+) cells [87] (Fig. 5). INK-ATTAC was first tested in BubR1^H/H^ progeroid mice: Treatment with AP substantially delayed the onset of age-related changes such as lordokyphosis and cataracts compared to untreated mice [87]. In an experiment more closely related to normal ageing, two cohorts of INK-ATTAC transgenic mice received treatment with AP or vehicle during 6 months starting at age 12 months (corresponding to approximately age 60 in humans). AP-treated mice had a 24–27% life span increase and delayed incidence and progression of cancer. Additional findings were markedly reduced glomerulosclerosis and increased tolerance to stress in the heart, attributed to a greater abundance of cardioprotective ATP-sensitive potassium (KATP) channels. Age-dependent behavioral changes such as diminished spontaneous and exploratory activity occurred more slowly in AP-treated mice [100]. Altogether, these results proved that naturally occurring senescent cells shorten healthy life span by promoting age-dependent changes in vital organs. The results were independent of sex and genetic background.

The first step in the development of small-molecule senolytic agents was conceiving the hypothesis that interfering with the anti-apoptotic, pro-survival mechanisms of senescent cells could achieve their selective elimination. The comparison of gene expression profiles between senescent and non-senescent cells revealed an overexpression of anti-apoptotic genes in the former. By targeting these anti-apoptotic factors in an RNAi screen, Zhu and colleagues identified six specific mediators (including EFNB1 and 3, p21, PI3KCD, BCL-xl, and PAI-2) whose knock-down triggered selective death of senescent preadipocytes and endothelial cells [101]. Small-molecule drugs targeting these products have continued to be developed as senolytics: dasatinib, an EphA2 receptor inhibitor [102], and quercetin, a kinase-inhibiting flavonol [103].

## How Can We Fight Cellular Senescence?

**Fig. 6:**
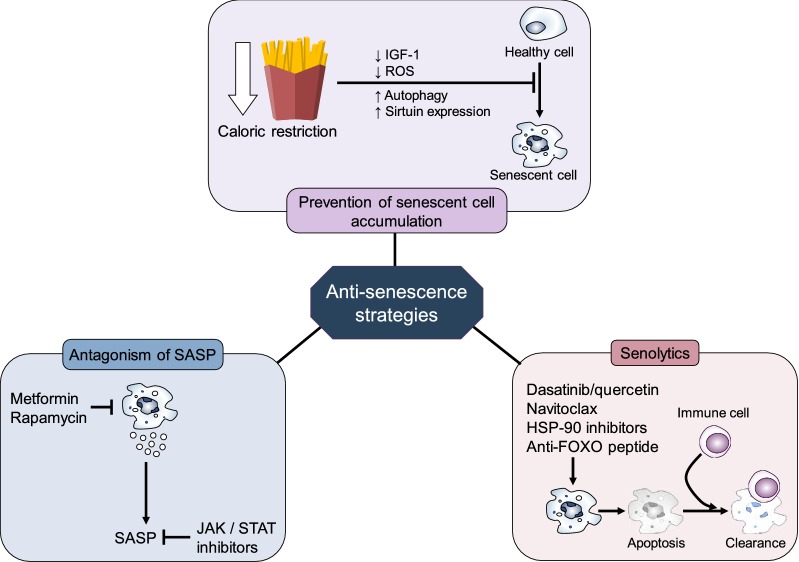
Therapeutic strategies to counter the effects of senescent cells. IGF-1 insulin-like growth factor 1, ROS reactive oxygen species, SASP senescence-associated secretory phenotype, JAK/STAT Janus kinase/signal transducers and activators of transcription, HSP-90 heat-shock protein 90, FOXO forkhead box proteins O

Strategies against cell senescence that can be translated into therapies for use in humans may be classified into the following groups: (1) non-pharmacological interventions that prevent the accumulation of senescent cells, (2) pharmacological therapies aimed at reducing the amount of SASP molecules produced by already existing senescent cells, and (3) pharmacological therapies aimed at reducing the number of senescent cells in the organism (true senolytics) (Fig. 6). The challenge with the second strategy lies in the ability to block the deleterious effects of SASP proteins, without interfering with their anti-oncogenic properties.

Within the first group, studies in mice have shown that a 3-month, 26% calorically restricted diet started at adulthood can reduce the number of senescent cells in rapidly proliferating (intestinal) and slowly proliferating (hepatic) tissues [104]. A study of the human colonic mucosa showed that adults who had been voluntarily exposed to a 30% caloric restriction (with appropriate nutrition) for over 10 years had significantly reduced expression of p16^INK4a^ and reduced local concentrations of IL-6 [105]. A recent study in mice demonstrated that calorie restriction, even if started at adulthood, prevents fat deposition and the concomitant increase in telomere-associated DNA damage foci (TAF) in hepatocytes [106]. The spectrum of cell senescence has been widened by studies showing that post-mitotic neurons may also display a senescent phenotype that can be ameliorated by a hypocaloric diet [107]. The mechanisms by which caloric restriction may reduce the appearance of new senescent cells include a reduction of ROS from nutrient metabolism, stimulation of autophagy, increased expression of sirtuins, and enhancement of normal DDR mechanisms [108]. At the organismal level, caloric restriction leads to reductions in bioavailable IGF-1 [109], a known inducer of cellular senescence over the long term [66].

The category of anti-SASP compounds contains mostly molecules approved for other indications and that have demonstrated anti-senescence properties in vitro, in animal models, or in translational studies in humans. Among them is the antidiabetic metformin, which is able to block the SASP in transformed fibroblasts [110]. Chronic metformin use is associated with extended life span and health span, independent of its antihyperglycemic efficacy [111]. Another long-time used medication with anti-SASP properties is the immunosuppressant rapamycin (sirolimus). By binding to the intracellular signaling molecule mammalian target of rapamycin (mTOR), the drug inhibits transcription of several SASP members and increases autophagy of senescent cells [112, 113]. A third group of approved drugs with anti-SASP features is JAK-STAT inhibitors. The JAK-STAT (signal transductor and activator of transcription) pathway is an intracellular signaling cascade responsible for many pro-inflammatory responses. Inhibition of this pathway has an anti-SASP effect in vitro [114]. Since several JAK inhibitors are available for the treatment of autoimmune diseases [115], a natural extension would be to assess their potential against cell senescence in humans.

Most efforts, however, have been placed in the development of senolytics, compounds with the potential to eliminate or reduce the numeric burden of senescent cells. Compared to the blockade of SASP, this seems to be a more efficient strategy, as in theory it would only need to be used sporadically in order to eliminate the senescent cells accumulated over a period of time.

### Dasatinib and Quercetin

Dasatinib is a tyrosine kinase inhibitor currently approved for the treatment of chronic myelogenous leukemia. Dasatinib and the natural polyphenolic antioxidant quercetin inhibit key transcriptional nodes used by senescent cells for survival [101]. The combination of dasatinib + quercetin greatly reduced senescent cells in mice with radiation exposure, increased DNA damage, or, most importantly, chronological ageing. In the Ercc1^−/Δ^ mouse model of accelerated ageing, dasatinib + quercetin successfully prolonged health span and life span [101]. Likewise, the combination reduces the development of complications in diet-induced obese mice [116]. Later studies from the same group found that the cocktail also prevents the accumulation of senescent cells in human adipose tissue explants, and extends the health span and life span of naturally aged mice [117]. In a proof-of-concept, open label, pilot study including nine patients with diabetic nephropathy, a 3-day course of 100 mg dasatinib + 1000 mg quercetin/day significantly reduced senescent cells and macrophage infiltration in adipose tissue at 11 days post-treatment [118]. Plasma levels of various SASP interleukins and metalloproteinases were also reduced. Planned or ongoing clinical trials will assess the effect of this senolytic combination on surrogate outcomes in patients with Alzheimer’s disease (clinicaltrials.gov ID NCT04063124) or chronic kidney disease (clinicaltrials.gov ID NCT02848131).

### Bcl Family Inhibitors

The Bcl family comprises a group of cell cycle regulatory proteins, some of which have anti-apoptotic effects, while others have pro-apoptotic effects. Navitoclax is an anticancer drug that acts by inhibiting several Bcl family members and promoting the release of pro-apoptotic factors. In contrast to dasatinib + quercetin, navitoclax lacks senolytic effect in adipose tissue, but displays a strong effect on endothelial cells and fibroblasts [119]. This molecule also reduces the burden of senescent cells in mice exposed to radiation [120] or chemotherapy [91].

### HSP90 Inhibitors

Geldanamycin and tanespimycin are inhibitors of the chaperone HSP90 and were recently identified as senolytics using an SA-β-Gal activity-based screening of compounds regulating autophagy in Ercc1^−/−^ murine embryonic fibroblasts [121]. Administration of 17-DMAG (alvespimycin), a water-soluble derivative of geldanamycin, to progeroid Ercc1^−/Δ^ mice significantly reduced a composite score of age-associated abnormalities including kyphosis, dystonia, tremor, grip strength, ataxia, gait disorder, and overall body condition. The drug attenuated expression of p16^INK4A^ in the kidney, but not in the liver of treated animals.

### Forkhead Box Proteins O (FOXO)-Targeting Peptide

Transcription factors of the FOXO family have been implicated in processes related to IGF-1, cell ageing, and senescence [122]. A peptide capable of preventing the FOXO4/p53 interaction excludes p53 from the nucleus and causes preferential apoptosis of senescent cells. The anti-FOXO peptide reduced the burden of cell senescence and toxicity caused by exposure of mice to doxorubicin, and improved physical fitness and renal function in progeroid and naturally aged mice [123].

### Fisetin

Fisetin is a polyphenolic compound naturally present in multiple fruits and vegetables. In an in vitro screen of ten natural flavonoids for senolytic activity, fisetin came out as the strongest compound. The screen was based on SA-β-Gal positivity after exposure to each compound in two cell types: Primary murine embryonic fibroblasts induced to senescence through oxidative stress (20% O_2_) and human fibroblasts induced to senescence through exposure to etoposide [124]. Administration of a diet with 500 ppm fisetin to Ercc1^−/Δ^ progeroid mice for 10 weeks reduced the tissue expression of p16^Ink4a^, p21^Cip1^, and most SASP factors. Similar results were found in naturally aged mice and human adipose tissue explants [124].

### Immunotherapies

A different emerging approach involves the enhancement of the natural capacity of immune cells to clear senescent cells. Examples of these strategies include the autologous transplantation of immune cells after being challenged ex vivo with senescent cell-specific antigens, or the use of antibodies that target senescent cells for removal by natural killer cells [125].

## Conclusion

The successful development of senolytics for clinical use will allow us to target multiple age-related conditions including cancer, atherosclerosis, metabolic syndrome/diabetes, immune dysfunction, neurodegeneration, neurovascular events, pulmonary fibrosis, osteoporosis, and impaired vision with a single agent or agent combination [126, 127]. Even in models of relatively rare autoimmune diseases like primary sclerosing cholangitis, inhibition of the SASP of senescent cholangiocytes through Bcl-2 inhibition reduced tissue damage and subsequent fibrosis [128]. Chronic non-communicable diseases are strongly associated with aging and constitute a top health priority for every country in the world. Senolytics offer the appealing prospect of simultaneously targeting the shared biological processes that underlie many of these diseases, extending life span and health span, and ushering into a new era of therapeutics. We believe that every intervention aimed at improving the quantity and quality of human life should be thoroughly explored and researched, but undoubtedly this promise entails very profound ethical and even philosophical questions. If health span and life span can be significantly extended, will our societies be ready for this demographic transition? How could we guarantee that the potential benefits of a senotherapeutic revolution can be accessible to most citizens, so that living better and longer does not become a privilege determined solely by economic status? These and other issues will gain further relevance over the coming years. Meanwhile, research on senolytics and other therapies against cell senescence continues at an accelerated pace.

## References

[1] Roser M, Ortiz-Ospina E, Ritchie H. Life expectancy. OurWorldInData.org. 2020. https://ourworldindata.org/life-expectancy. Accessed 19 Feb 2020.

[2] Mason A, Lee R, Abrigo M, Lee SH. Support ratios and demographic dividens: estimates for the world. United Nations, Department of Economic and Social Affairs, Population Division. Technical paper no. 2017/1. New York; 2017.

[3] World Health Organization. World health statistics overview 2019: monitoring health for SDG, sustainable development goals. License: CC BY-NC-SA 3.0 IGO. Geneva; 2019.

[4] World Health Organization. Global health observatory (GHO) data, top 10 cases of death. https://www.who.int/gho/mortality_burden_disease/causes_death/top_10/en/. Accessed 10 Jan 2020.

[5] Institute for Health and Evaluation (IHME) (2018). Findings from the global burden of disease study 2017.

[6] He S, Sharpless NE (2017). Senescence in health and disease. Cell.

[7] Shay JW, Wright WE (2000). Hayflick, his limit, and cellular ageing. Nat Rev Mol Cell Biol.

[8] Hayflick L, Moorhead PS (1961). The serial cultivation of human diploid cell strains. Exp Cell Res.

[9] Chen Q, Fischer A, Reagan JD, Yan LJ, Ames BN (1995). Oxidative DNA damage and senescence of human diploid fibroblast cells. Proc Natl Acad Sci USA.

[10] Serrano M, Lin AW, McCurrach ME, Beach D, Lowe SW (1997). Oncogenic ras provokes premature cell senescence associated with accumulation of p53 and p16INK4a. Cell.

[11] Lowe SW, Cepero E, Evan G (2004). Intrinsic tumour suppression. Nature.

[12] Nakagami H (2019). Cellular senescence and senescence-associated T cells as a potential therapeutic target. Geriatr Gerontol Int.

[13] Kang C (2019). Senolytics and senostatics: a two-pronged approach to target cellular senescence for delaying aging and age-related diseases. Mol Cells.

[14] Di Leonardo A, Linke SP, Clarkin K (1994). DNA damage triggers a prolonged p53-dependent G1 arrest and long-term induction of Cip1 in normal human fibroblasts. Genes Dev.

[15] Ogryzko VV, Hirai TH, Russanova VR (1996). Human fibroblast commitment to a senescence-like state in response to histone deacetylase inhibitors is cell cycle dependent. Mol Cell Biol.

[16] Terzi MY, Izmirli M, Gogebakan B (2016). The cell fate: senescence or quiescence. Mol Biol Rep.

[17] Patel PL, Suram A, Mirani N (2016). Derepression of hTERT gene expression promotes escape from oncogene-induced cellular senescence. Proc Natl Acad Sci USA.

[18] Milanovic M, Fan DNY, Belenki D (2018). Senescence-associated reprogramming promotes cancer stemness. Nature.

[19] Chen W, Kang J, Xia J (2008). p53-related apoptosis resistance and tumor suppression activity in UVB-induced premature senescent human skin fibroblasts. Int J Mol Med.

[20] Childs BG, Baker DJ, Kirkland JL (2014). Senescence and apoptosis: dueling or complementary cell fates?. EMBO Rep.

[21] Myrianthopoulos V, Evangelou K, Vasileiou PVS (2019). Senescence and senotherapeutics: a new field in cancer therapy. Pharmacol Ther.

[22] Salotti J, Johnson PF (2019). Regulation of senescence and the SASP by the transcription factor C/EBPβ. Exp Gerontol.

[23] Da Silva-Álvarez S, Picallos-Rabina P, Antelo-Iglesias L (2019). The development of cell senescence. Exp Gerontol.

[24] Acosta JC, O'Loghlen A, Banito A (2008). Chemokine signaling via the CXCR2 receptor reinforces senescence. Cell.

[25] Acosta JC, Banito A, Wuestefeld T (2013). A complex secretory program orchestrated by the inflammasome controls paracrine senescence. Nat Cell Biol.

[26] Demaria M, Ohtani N, Youssef SA (2014). An essential role for senescent cells in optimal wound healing through secretion of PDGF-AA. Dev Cell.

[27] Mosteiro L, Pantoja C, Alcazar N (2016). Tissue damage and senescence provide critical signals for cellular reprogramming in vivo. Science.

[28] Storer M, Mas A, Robert-Moreno A (2013). Senescence is a developmental mechanism that contributes to embryonic growth and patterning. Cell.

[29] Franceschi C, Bonafè M, Valensin S (2000). Inflamm-aging. An evolutionary perspective on immunosenescence. Ann N Y Acad Sci.

[30] López-Otín C, Blasco MA, Partridge L (2013). The hallmarks of aging. Cell.

[31] Valenzuela CA, Quintanilla R, Moore-Carrasco R (2017). The potential role of senescence as a modulator of platelets and tumorigenesis. Front Oncol.

[32] Parrinello S, Coppe JP, Krtolica A (2005). Stromal-epithelial interactions in aging and cancer: senescent fibroblasts alter epithelial cell differentiation. J Cell Sci.

[33] Angelini PD, Zacarias Fluck MF, Pedersen K (2013). Constitutive HER2 signaling promotes breast cancer metastasis through cellular senescence. Cancer Res.

[34] Herranz N, Gil J (2018). Mechanisms and functions of cellular senescence. J Clin Investig.

[35] van Deursen JM (2014). The role of senescent cells in ageing. Nature.

[36] Faget DV, Ren Q, Stewart SA (2019). Unmasking senescence: context-dependent effects of SASP in cancer. Nat Rev Cancer.

[37] Galbiati A, Beauséjour C, d’Adda di Fagagna F (2017). A novel single-cell method provides direct evidence persistent DNA damage in senescent cells and aged mammalian tissues. Aging Cell.

[38] Jackson S, Bartek J (2009). The DNA-damage response in human biology and disease. Nature.

[39] Rogakou E, Boon C, Redon C (1999). Megabase chromatin domains involved in DNA double-strand breaks in vivo. J Cell Biol.

[40] Sedelnikova OA, Horikawa I, Zimonjic DB (2004). Senescing human cells and ageing mice accumulate DNA lesions with unrepairable double-strand breaks. Nat Cell Biol.

[41] Alimonti A, Nardella C, Chen Z (2010). A novel type of cellular senescence that can be enhanced in mouse models and human tumor xenografts to suppress prostate tumorigenesis. J Clin Investig.

[42] Demidenko Z, Blagosklonny M (2008). Growth stimulation leads to cellular senescence when the cell cycle is blocked. Cell Cycle.

[43] Shenghui H, Sharpless N (2017). Senescence in health and disease. Cell.

[44] Pluquet O, Pourtier A, Abbadie C (2014). The unfolded protein response and cellular senescence. A review in the theme: cellular mechanisms of endoplasmic reticulum stress signaling in health and disease. Am J Physiol Cell Physiol.

[45] Burton D, Faragher R (2018). Obesity and type-2 diabetes as inducers of premature cellular senescence and ageing. Biogerontology.

[46] Minamino T, Orimo M, Shimizu I (2009). A crucial role for adipose tissue p53 in the regulation of insulin resistance. Nat Med.

[47] Higuchi M, Dusting G, Peshavariya H (2013). Differentiation of human adipose-derived stem cells into fat involves reactive oxygen species and forkhead box-O1 mediated upregulation of antioxidant enzymes. Stem Cells Dev.

[48] Dodig S, Cepelak I, Pavic I (2019). Hallmarks of senescence and aging. Biochem Med (Zagreb).

[49] Muñoz-Espín D, Serrano M (2014). Cellular senescence: from physiology to pathology. Nat Rev Mol Cell Biol.

[50] Wang E, Gundersen D (1984). Increased organization of cytoskeleton accompanying the aging of human fibroblasts in vitro. Exp Cell Res.

[51] Nishio K, Inoue A, Qiao S (2001). Senescence and cytoskeleton: overproduction of vimentin induces senescent-like morphology in human fibroblasts. Histochem Cell Biol.

[52] Ogrodnik M, Salmonowicz H, Jurk D (2019). Expansion and cell-cycle arrest: common denominators of cellular senescence. Trends Biochem Sci.

[53] Kurz DJ, Decary S, Hong Y (2000). Senescence-associated (beta)-galactosidase reflects an increase in lysosomal mass during replicative ageing of human endothelial cells. J Cell Sci.

[54] Rose-John S (2018). Interleukin-6 family cytokines. Cold Spring Harb Perspect Biol.

[55] Jones SA, Scheller J, Rose-John S (2011). Therapeutic strategies for the clinical blockade of IL-6/gp130 signaling. J Clin Investig.

[56] Kuilman T, Michaloglou C, Vredeveld LC (2008). Oncogene-induced senescence relayed by an interleukin-dependent inflammatory network. Cell.

[57] Kumar S, Millis AJ, Baglioni C (1992). Expression of interleukin 1-inducible genes and production of interleukin 1 by aging human fibroblasts. Proc Natl Acad Sci USA.

[58] Orjalo AV, Bhaumik D, Gengler BK (2009). Cell surface-bound IL-1alpha is an upstream regulator of the senescence-associated IL-6/IL-8 cytokine network. Proc Natl Acad Sci USA.

[59] Zlotnik A, Yoshie O (2000). Chemokines: a new classification system and their role in immunity. Immunity.

[60] Coppé JP, Desprez PY, Krtolica A (2010). The senescence-associated secretory phenotype: the dark side of tumor suppression. Annu Rev Pathol.

[61] Chen J, Mo R, Lescure PA (2003). Aging is associated with increased T-cell chemokine expression in C57BL/6 mice. J Gerontol A Biol Sci Med Sci.

[62] Sasaki M, Miyakoshi M, Sato Y (2014). Chemokine–chemokine receptor CCL2–CCR2 and CX3CL1–CX3CR1 axis may play a role in the aggravated inflammation in primary biliary cirrhosis. Dig Dis Sci.

[63] Bachstetter AD, Morganti JM, Jernberg J (2011). Fractalkine and CX 3 CR1 regulate hippocampal neurogenesis in adult and aged rats. Neurobiol Aging.

[64] Mecca C, Giambanco I, Donato R (2018). Microglia and aging: the role of the TREM2–DAP12 and CX3CL1–CX3CR1 axes. Int J Mol Sci.

[65] Pollak M (2008). Insulin and insulin-like growth factor signaling in neoplasia. Nat Rev Cancer.

[66] Tran D, Bergholz J, Zhang H (2014). Insulin-like growth factor-1 regulates the SIRT1-p53 pathway in cellular senescence. Aging Cell.

[67] Lopes-Paciencia S, Saint-Germain E, Rowell MC (2019). The senescence-associated secretory phenotype and its regulation. Cytokine.

[68] Bonnans C, Chou J, Werb Z (2014). Remodelling the extracellular matrix in development and disease. Nat Rev Mol Cell Biol.

[69] West MD, Pereira-Smith OM, Smith JR (1989). Replicative senescence of human skin fibroblasts correlates with a loss of regulation and overexpression of collagenase activity. Exp Cell Res.

[70] Millis AJ, Hoyle M, McCue HM (1992). Differential expression of metalloproteinase and tissue inhibitor of metalloproteinase genes in aged human fibroblasts. Exp Cell Res.

[71] Hassona Y, Cirillo N, Prime HK (2014). Senescent cancer-associated fibroblasts secrete active MMP-2 that promotes keratinocyte dis-cohesion and invasion. Br J Cancer.

[72] Özcan S, Alessio N, Acar MB (2016). Unbiased analysis of senescence associated secretory phenotype (SASP) to identify common components following different genotoxic stresses. Aging (Albany NY).

[73] Zeng G, Millis AJ (1996). Differential regulation of collagenase and stromelysin mRNA in late passage cultures of human fibroblasts. Exp Cell Res.

[74] Quillard T, Araújo HA, Franck G (2014). Matrix metalloproteinase-13 predominates over matrix metalloproteinase-8 as the functional interstitial collagenase in mouse atheromata. Arterioscler Thromb Vasc Biol.

[75] McQuibban GA, Gong JH, Wong JP (2002). Matrix metalloproteinase processing of monocyte chemoattractant proteins generates CC chemokine receptor antagonists with anti-inflammatory properties in vivo. Blood.

[76] Hernandez-Segura A, Nehme J, Demaria M (2018). Hallmarks of cellular senescence. Trends Cell Biol.

[77] Rayess H, Wang M, Srivatsan E (2011). Cellular senescence and tumor suppressor gene. Int J Cancer.

[78] Karimian A, Ahmadi Y, Yousefi B (2016). Multiple functions of P21 in cell cycle, apoptosis and transcriptional regulation after DNA damage. DNA Repair (Amst).

[79] Kirkland J, Tchkonia T (2017). Cellular senescence: a translational perspective. EBioMedicine.

[80] Kobbe V (2018). Cellular senescence: a view throughout organismal life. Cell Mol Life Sci.

[81] Muñoz-Espín D, Cañamero M, Maraver A (2013). Programmed cell senescence during mammalian embryonic development. Cell.

[82] Vicente R, Mausset-Bonnefont A, Jorgensen C (2016). Cellular senescence impact on immune cell fate and function. Aging Cell.

[83] Rajagopalan S (2014). HLA-G-mediated NK cell senescence promotes vascular remodeling: implications for reproduction. Cell Mol Immunol.

[84] Rajagopalan S, Long E (2012). Cellular senescence induced by CD158d reprograms natural killer cells to promote vascular remodeling. Proc Natl Acad Sci USA.

[85] Stein GH, Drullinger LF, Soulard A (1999). Differential roles for cyclin-dependent kinase inhibitors p21 and p16 in the mechanisms of senescence and differentiation in human fibroblasts. Mol Cell Biol.

[86] Jun J-I, Lau L (2010). The matricellular protein CCN1 induces fibroblast senescence and restricts fibrosis in cutaneous wound healing. Nat Cell Biol.

[87] Baker D, Wijshake T, Tchkonia T (2011). Clearance of p16Ink4a-positive senescent cells delays ageing-associated disorders. Nature.

[88] Childs B, Baker D, Wijshake T (2016). Senescent intimal foam cells are deleterious at all stages of atherosclerosis. Science.

[89] Pal A, Potjer T, Thomsen S (2016). Loss of function mutations in the cell-cycle control gene CDKN2A impact on glucose homeostasis in humans. Diabetes.

[90] González-Navarro H, Vinue A, Sanz MJ (2013). Increased dosage of Ink4/Arf protects against glucose intolerance and insulin resistance associated with aging. Aging Cell.

[91] Demaria M, O’Leary MN, Chang J (2017). Cellular senescence promotes adverse effects of chemotherapy and cancer relapse. Cancer Discov.

[92] Calcinotto A, Kohli J, Zagato E (2019). Cellular senescence: aging, cancer, and injury. Physiol Rev.

[93] Bhat R, Crowe E, Bitto A (2012). Astrocyte senescence as a component of Alzheimer’s disease. PLoS One.

[94] Chinta SJ, Woods G, Demaria M (2017). Cellular senescence is induced by the environmental neurotoxin paraquat and contributes to neuropathology linked to Parkinson’s disease. Cell Rep.

[95] Courtois-Cox S, Jones SL, Cichowski K (2008). Many roads lead to oncogene-induced senescence. Oncogene.

[96] Childs BG, Gluscevic M, Baker DJ (2017). Senescent cells: an emerging target for diseases of ageing. Nat Rev Drug Discov.

[97] Trujillo M, Pajvani U, Scherer P (2005). Apoptosis through targeted activation of caspase (“ATTAC-mice”): novel mouse models of inducible and reversible tissue ablation. Cell Cycle.

[98] Clackson T, Yang W, Rozamus L (1998). Redesigning an FKBP–ligand interface to generate chemical dimerizers with novel specificity. Proc Natl Acad Sci USA.

[99] Wang W, Wu J, Zhang Z, Tong T (2001). Characterization of regulatory elements on the promoter region of p16INK4a that contribute to overexpression of p16 in senescent fibroblasts. J Biol Chem.

[100] Baker DJ, Childs B, Durik M (2016). Naturally occurring p16Ink4a-positive cells shorten healthy lifespan. Nature.

[101] Zhu Y, Tchkonia T, Pirtskhalava T (2015). The Achilles' heel of senescent cells: from transcriptome to senolytic drugs. Aging Cell.

[102] Chang Q, Jorgensen C, Pawson T (2008). Hedley DW Effects of dasatinib on EphA2 receptor tyrosine kinase activity and downstream signalling in pancreatic cancer. Br J Cancer.

[103] Boly R, Gras T, Lamkami T (2011). Quercetin inhibits a large panel of kinases implicated in cell biology. Int J Oncol.

[104] Wang C, Maddick M, Miwa S (2010). Adult-onset, short-term dietary restriction reduces cell senescence in mice. Aging (Albany NY).

[105] Fontana L, Mitchell SE, Wang B (2018). The effects of graded caloric restriction: XII. Comparison of mouse to human impact on cellular senescence in the colon. Aging Cell.

[106] Ogrodnik M, Miwa S, Tchkonia T (2017). Cellular senescence drives age-dependent hepatic steatosis. Nat Commun.

[107] Jurk D, Wang C, Miwa S (2012). Postmitotic neurons develop a p21-dependent senescence-like phenotype driven by a DNA damage response. Aging Cell.

[108] Fontana L, Nehme J, Demaria M (2018). Caloric restriction and cellular senescence. Mech Ageing Dev.

[109] Fontana L, Villareal DT, Das SK (2016). Effects of 2-year calorie restriction on circulating levels of IGF-1, IGF-binding proteins and cortisol in nonobese men and women: a randomized clinical trial. Aging Cell.

[110] Moiseeva O, Deschênes-Simard X, St-Germain E (2013). Metformin inhibits the senescence-associated secretory phenotype by interfering with IKK/NF-κB activation. Aging Cell.

[111] Campbell JM, Bellman SM, Stephenson MD (2017). Metformin reduces all-cause mortality and diseases of ageing independent of its effect on diabetes control: a systematic review and meta-analysis. Ageing Res Rev.

[112] Wang R, Yu Z, Sunchu B, Shoaf J (2017). Rapamycin inhibits the secretory phenotype of senescent cells by a Nrf2-independent mechanism. Aging Cell.

[113] Gurău F, Baldoni S, Prattichizzo F (2018). Anti-senescence compounds: a potential nutraceutical approach to healthy aging. Ageing Res Rev.

[114] Xu M, Tchkonia T, Ding H (2015). JAK inhibition alleviates the cellular senescence-associated secretory phenotype and frailty in old age. Proc Natl Acad Sci USA.

[115] Roskoski R (2016). Janus kinase (JAK) inhibitors in the treatment of inflammatory and neoplastic diseases. Pharmacol Res.

[116] Palmer AK, Xu M, Zhu Y (2019). Targeting senescent cells alleviates obesity-induced metabolic dysfunction. Aging Cell.

[117] Xu M, Pirtskhalava T, Farr JN (2018). Senolytics improve physical function and increase lifespan in old age. Nat Med.

[118] Hickson LJ, Langhi Prata LGP, Bobart SA (2019). Senolytics decrease senescent cells in humans: preliminary report from a clinical trial of dasatinib plus quercetin in individuals with diabetic kidney disease. EBioMedicine.

[119] Zhu Y, Tchkonia T, Fuhrmann-Stroissnigg H (2016). Identification of a novel senolytic agent, navitoclax, targeting the Bcl-2 family of anti-apoptotic factors. Aging Cell.

[120] Chang J, Wang Y, Shao L (2016). Clearance of senescent cells by ABT263 rejuvenates aged hematopoietic stem cells in mice. Nat Med.

[121] Fuhrmann-Stroissnigg H, Ling YY, Zhao J (2017). Identification of HSP90 inhibitors as a novel class of senolytics. Nat Commun.

[122] Bourgeois B, Madl T (2018). Regulation of cellular senescence via the FOXO4-p53 axis. FEBS Lett.

[123] Baar MP, Brandt RM, Putavet DA (2017). Targeted apoptosis of senescent cells restores tissue homeostasis in response to chemotoxicity and aging. Cell.

[124] Yousefzadeh MJ, Zhu Y, McGowan SJ (2018). Fisetin is a senotherapeutic that extends health and lifespan. EBioMedicine.

[125] Prata LGPL, Ovsyannikova IG, Tchkonia T, Kirkland JL (2018). Senescent cell clearance by the immune system: emerging therapeutic opportunities. Semin Immunol.

[126] Khosla S, Farr JN, Kirkland JL (2018). Inhibiting cellular senescence: a new therapeutic paradigm for age-related osteoporosis. J Clin Endocrinol Metab.

[127] Campisi J, Kapahi P, Lithgow GJ, Melov S, Newman JC, Verdin E (2019). From discoveries in ageing research to therapeutics for healthy ageing. Nature.

[128] Sikora E, Bielak-Zmijewska A, Mosieniak G (2019). Targeting normal and cancer senescent cells as a strategy of senotherapy. Ageing Res Rev.

